# External validation of the Briganti 2019 nomogram to identify candidates for extended pelvic lymph node dissection among patients with high-risk clinically localized prostate cancer

**DOI:** 10.1007/s10147-021-01954-4

**Published:** 2021-06-12

**Authors:** Eri Fukagawa, Shinya Yamamoto, Sachiko Ohde, Kasumi Kaneko Yoshitomi, Kosuke Hamada, Yusuke Yoneoka, Motohiro Fujiwara, Ryo Fujiwara, Tomohiko Oguchi, Yoshinobu Komai, Noboru Numao, Takeshi Yuasa, Iwao Fukui, Junji Yonese

**Affiliations:** 1 Department of Urology, Cancer Institute Hospital, Japanese Foundation for Cancer Research, 3-8-31 Ariake, Koto-ku, Tokyo, 135-8550 Japan; 2 Graduate School of Public Health, St. Luke’s International University, 10-1 Akashi-cho, Chuo-ku, Tokyo, 104-0044 Japan

**Keywords:** External validation, Lymph node invasion, Nomogram, Prostate cancer, Radical prostatectomy

## Abstract

**Background:**

We aimed to establish an external validation of the Briganti 2019 nomogram in a Japanese cohort to preoperatively evaluate the probability of lymph node invasion in patients with high-risk, clinically localized prostate cancer.

**Methods:**

The cohort consisted of 278 patients with prostate cancer diagnosed using magnetic resonance imaging-targeted biopsy who underwent radical prostatectomy and extended pelvic lymph node dissection from 2012 to 2020. Patients were rated using the Briganti 2019 nomogram, which evaluates the probability of lymph node invasion. We used the area under curve of the receiver operating characteristic analysis to quantify the accuracy of the nomogram.

**Results:**

Nineteen (6.8%) patients had lymph node invasion. The median number of lymph nodes removed was 18. The area under the curve for the Briganti 2019 was 0.71. When the cutoff was set at 7%, 84 (30.2%) patients with extended pelvic lymph node dissection could be omitted, and only 1 (1.2%) patient with lymph node invasion would be missed. Sensitivity, specificity, and negative predictive values at the 7% cutoff were 94.7, 32.0, and 98.8%, respectively.

**Conclusion:**

This external validation showed that the Briganti 2019 nomogram was accurate, although there may still be scope for individual adjustments.

## Introduction

Pelvic lymph node dissection (PLND) represents the gold standard for detecting occult lymph node invasion (LNI) and confirming accurate staging of high-risk prostate cancer [1]. However, its therapeutic role, indication, and the extent of PLND required remain controversial [2, 3]. Moreover, there are complications, such as lymphocele, lymphedema, neurovascular injury, and thromboembolic events associated with PLND [4, 5]. Furthermore, PLND has additional attendant costs and requires extended operative time [4]. In the current era of widespread prostate-specific antigen (PSA)-based population screening, the actual number of cases with LNI is small, compared to the number of PLNDs performed. It is, therefore, of great importance to focus on the indications for PLNDs to avoid unnecessary invasion and the associated complications.

To achieve an ideal strategy that minimizes unnecessary invasion without missing progression, various nomograms have been developed and updated to preoperatively evaluate the risk of LNI and to determine the indications for PLND. The European Association of Urology (EAU) guidelines introduced Briganti, Partin, and Memorial Sloan Kettering Cancer Center (MSKCC) nomograms to estimate the risk of nodal metastases [1], whereas Japanese guidelines include Partin as well as the Japan Prostate Cancer (PC) table designed for Japanese patients [6]. All these nomograms are based on preoperative serum PSA, clinical tumor stage, and histopathological biopsy results, with some modification of each.

An updated version of the Briganti nomogram (Briganti 2019), which includes multiparametric-magnetic resonance imaging (MRI) findings and MRI-targeted biopsy results as parameters, was published in 2019 [7] and is considered to possibly be the "clinically most effective tool to date," noting that a full external validation is still outstanding in the EAU 2020 updated guidelines [8].

There are several techniques of MRI-targeted biopsy: cognitive fusion biopsy, fusion MRI-ultrasound-guided biopsy, and MRI-guided in-bore biopsy [9–12]. The superiority of the detection of clinically significant prostate cancer (csPC) by using a combined method of MRI-targeted and systematic biopsy compared with each single method has been reported [13]. As the diagnostic imaging technique improves, MRI-targeted biopsy has been more popular worldwide to achieve more accurate diagnoses.

We introduced MRI-targeted prostate biopsy in 2012. The indication for PLND had traditionally been dependent on the surgeon's decision, but since the publication of the Briganti 2019 nomogram, we have used this nomogram to identify candidates for PLND.

We aimed to externally validate the Briganti 2019 nomogram in a Japanese cohort of patients with prostate cancer to assess its accuracy in real-world clinical practice.

## Materials and methods

### Study cohort

Clinical and pathological data were retrospectively collected for patients diagnosed with prostate cancer using MRI-targeted biopsy and treated with radical prostatectomy (RP) with ePLND at the Cancer Institute Hospital, Japanese Foundation for Cancer Research, Tokyo, Japan from 2012 to 2020. During the period, RP was performed in 1155 patients. Patients were excluded from the study if their malignancies were only confirmed by systematic biopsy (i.e., they did not undergo targeted biopsy or obtained a negative targeted biopsy result), if they underwent biopsy at another institution, received neoadjuvant hormone or radiation therapy, or underwent RP without ePLND. Hence, the remaining 278 patients were included in the current study. The determination of serum PSA level and pelvic multi-parametric MRI were performed prior to the biopsy. MRI scans were read by trained radiologists without the use of the Prostate Imaging Reporting & Data System (PI-RADS). Transrectal ultrasound-guided transperineal biopsy was combined with MRI-targeted (1–4 cores per lesion via cognitive registration) and systematic (8–14 cores; median 8 cores) methods, and the number of cores taken was decided by the treating surgeon. Imaging studies preoperatively confirmed that all patients had no metastases. Skilled surgeons performed RP and ePLND (including external, internal iliac, and obturator lymph nodes) using either an open or minimum incision endoscopic or robot-assisted approach [14, 15]. The probability of LNI was evaluated using the Briganti 2019 nomogram [7], Briganti 2017 nomogram [16], Briganti 2012 nomogram [17], MSKCC nomogram [18], Partin 2017 nomogram [19], and Japan PC table [20], based on clinical data and biopsy results (Table 1). This study was approved by the Institutional Review Board at the Cancer Institute Hospital, Japanese Foundation for Cancer Research (IRB No. 2020-1198).

**Table 1:** Variables included in nomograms predicting lymph node invasion at radical prostatectomy in patients with prostate cancer

Nomogram	Covariates
Briganti 2019^†^ [7]	PSA at diagnosis
	Clinical stage on multiparametric MRI
	Grade group on MRI-targeted biopsy
	Maximum diameter of the index lesion on multiparametric MRI
	Percentage of positive cores with clinically significant cancer on systematic biopsy
Briganti 2017^‡^ [16]	Biopsy Gleason grade group
	Clinical stage
	Preoperative PSA
	Percentage of positive cores with the highest-grade disease
	Percentage of positive cores with the lower-grade disease
Briganti 2012^§^ [17]	PSA at diagnosis
	Clinical stage
	Primary Gleason grade
	Secondary Gleason grade
	Percentage of positive cores
MSKCC^¶^ [18]	Preoperative PSA
	Primary biopsy Gleason grade
	Secondary biopsy Gleason grade
	Clinical stage
	Number of negative cores
	Number of positive cores
Partin 2017^††^ [19]	Preoperative PSA (0–4, 4.1–6, 6.1–10 and greater than 10 ng/ml)
	Gleason score (5–6, 3 + 4, 4 + 3/8, and 9–10)
	Clinical stage (T1c, T2a and T2b/T2c)
Japan PC table^‡‡^ [20]	Preoperative PSA (0–4, 4.1–6, 6.1–8, 8.1–10, and greater than 10 ng/ml)
	Gleason score (6, 3 + 4, 4 + 3, and 8–10)
	Clinical stage (T1c, T2a, T2b, and T2c)

### Statistical analyses

Data were summarized, including all of the parameters of each of the six nomograms. Frequencies and percentages were determined for categorical variables, and medians and ranges for continuous variables. Comparisons between the groups with and without histologically confirmed LNI were performed using Fisher’s exact test for qualitative variables and the Mann–Whitney *U* test for quantitative variables. The area under the curve (AUC) of the receiver operating characteristic (ROC) analysis was obtained to quantify the accuracy of each nomogram. The calibration plot representing the relationship between the predictive probabilities calculated with the Briganti 2019 coefficients on the *x*-axis and the observed frequencies on the *y*-axis was also studied. All statistical tests were two-sided with the significance level set at *P* < 0.05. Analyses were conducted using R version 3.6.3 (The R Foundation for Statistical Computing, Vienna, Austria).

## Results

### Baseline characteristics

Descriptive patient characteristics are shown in Table 2. Overall, 19 (6.8%) patients exhibited LNI at final pathology. The median number of lymph nodes removed was 18 (5–55). Among patients with and without LNI, there were statistically significant differences in preoperative PSA, Gleason grade group in overall/MRI-targeted/systematic biopsy, primary and secondary Gleason grade overall, percentage positive cores on overall/systematic biopsy, percentage of positive cores with lower-grade taken overall, as well as the number of positive and negative cores taken overall (*P* < 0.05). Pathological characteristics are also shown in Table 2, which achieved statistical significance in the Gleason grade group regarding surgical specimens and pathological T stage.

**Table 2:** Descriptive statistics for patients with clinically localized prostate cancer diagnosed via MRI-targeted biopsy and treated with RP and ePLND

Parameter	Comparison within the external validation cohort							Development cohort				
	Overall		pN0		pN1		*P* value	pN0		pN1		*P* value
Patients, *n* (%)	278	(100)	259	(93.2)	19	(6.8)		435	(87.5)	62	(12.5)	
Median age at surgery, years (IQR)	67	(63–71)	67	(63–71)	69	(65–71)	0.364	65	(60–70)	64	(60–71)	0.8
Median preoperative PSA, ng/ml (IQR)^†,‡,§, ¶, ††,‡‡^	8.2	(5.7–12)	8.0	(5.6–11)	11.4	(10–15)	0.013	7.2	(5.1–11)	11	(6.7–21)	< 0.001
Median prostate volume, ml (IQR)	23.6	(19–30)	23.5	(19–29)	25.9	(20–31)	0.511	43	(33–55)	48	(34–59)	0.1
Median maximum index lesion diameter on mpMRI, mm (IQR)^†^	10	(8–14)	10	(8–14)	13	(10–18)	0.157	10	(9–14)	15	(10–18)	< 0.001
Clinical stage on mpMRI, *n* (%)^†,‡,§, ¶,††,‡‡^												
Organ-confined	207	(75)	195	(75)	12	(63)	0.377	358	(85)	29	(47)	< 0.001
Extracapsular extension	66	(24)	59	(23)	7	(37)		49	(12)	19	(31)	
Seminal vesicle invasion	5	(1.8)	5	(1.9)	0	(0.0)		13	(3)	14	(22)	
Biopsy grade roup overall, *n* (%)^‡,††,‡‡^												
1	6	(2.2)	6	(2.3)	0	(0.0)	0.005	55	(13)	1	(2)	< 0.001
2	37	(13)	36	(14)	1	(5.3)		236	(54)	15	(24)	
3	73	(26)	71	(27)	2	(11)		78	(18)	16	(26)	
4	92	(33)	86	(33)	6	(32)		45	(10)	15	(24)	
5	70	(25)	60	(23)	10	(53)		21	(5)	15	(24)	
Primary Gleason grade overall^§,¶^												
3	46	(16.5)	45	(17.4)	1	(5.3)	0.020					
4	211	(75.9)	197	(76.1)	14	(73.7)						
5	21	(7.6)	17	(6.6)	4	(21.1)						
Secondary Gleason grade overall^§,¶^												
3	79	(28.4)	77	(29.7)	2	(10.5)	0.036					
4	138	(49.6)	128	(49.4)	10	(52.6)						
5	61	(21.9)	54	(20.8)	7	(36.8)						
Median cores taken overall, *n* (IQR)	15	(12–16)	15	(12–16)	16	(12–16)	0.828	16	(14–18)	16	(14–18)	0.2
Median positive cores taken overall, *n* (IQR)^¶^	5	(4–7)	5	(4–7)	8	(6–9)	0.006	5	(3–8)	5	(9–12)	< 0.001
Median negative cores taken overall, *n* (IQR)^¶^	8	(6–11)	8	(7–11)	7	(5–10)	0.029					
Median percentage positive cores overall, % (IQR)^‡^	39	(25–50)	38	(25–50)	56	(42–67)	0.005	33	(20–50)	55	(36–80)	< 0.001
Median positive cores with highest-grade disease, % (IQR)^‡^	13	(8–25)	13	(8–25)	13	(8–25)	0.434	20	(12–38)	40	(24–60)	< 0.001
Median positive cores with lower-grade disease, % (IQR)^‡^	19	(8–35)	19	(8–33)	31	(13–50)	0.021	16	(8–27)	21	(10–30)	0.1
Grade group on MRI-targeted biopsy, *n* (%)^†^												
1	15	(5.4)	15	(5.8)	0	(0.0)	0.025	72	(17)	1	(2)	< 0.001
2	58	(21)	56	(22)	2	(11)		225	(52)	15	(24)	
3	74	(27)	69	(27)	5	(26)		72	(17)	16	(26)	
4	73	(26)	69	(27)	4	(21)		46	(11)	17	(27)	
5	58	(21)	50	(19)	8	(42)		20	(5)	13	(21)	
Target-cores taken on MRI-targeted biopsy, *n* (%)												
2 ≥	72	(26)	68	(26)	4	(21)	0.663	165	(38)	27	(44)	0.1
3	25	(9.0)	24	(9.3)	1	(5.3)		94	(22)	18	(29)	
4	145	(52)	133	(51)	12	(63)		77	(18)	10	(16)	
≥ 5	36	(13)	34	(13)	2	(11)		99	(23)	7	(11)	
Positive cores on MRI-targeted biopsy, *n* (%)												
1	56	(20)	52	(20)	4	(21)	0.121	111	(26)	5	(8.1)	0.1
2	87	(31)	84	(32)	3	(16)		173	(40)	32	(51)	
3	58	(21)	56	(22)	2	(11)		69	(16)	16	(26)	
≥4	77	(28)	67	(26)	10	(53)		82	(18)	9	(15)	
Grade group on systematic biopsy, *n* (%)												
Negative	28	(10)	28	(11)	0	(0.0)	0.003	80	(18)	4	(7)	< 0.001
1	23	(8.3)	23	(8.9)	0	(0.0)		100	(23)	6	(10)	
2	63	(23)	60	(23)	3	(16)		171	(40)	14	(23)	
3	57	(21)	55	(21)	2	(11)		44	(10)	15	(24)	
4	62	(22)	56	(22)	6	(32)		25	(6)	9	(15)	
5	45	(16)	37	(14)	8	(42)		15	(4)	14	(23)	
Median systematic cores taken, *n* (IQR)	8	(8–14)	8	(8–14)	8	(8–14)	0.917	12	(10–15)	12	(10–16)	0.2
Median cores with csPC on systematic biopsy, % (IQR)^†^	25	(13–38)	25	(13–38)	38	(32–50)	0.005	12	(0–37)	42	(17–76)	< 0.001
Surgical technique, *n* (%)												
Open RP	106	(38)	97	(38)	9	(47)	0.68	40	(9.2)	3	(4.8)	0.2
Minimum incision endoscopic RP	7	(2.5)	7	(2.7)	0	(0.0)		0	(0)	0	(0)	
Robot-assisted RP	165	(59)	155	(60)	10	(53)		395	(90)	59	(95)	
Gleason grade group on final pathology, *n* (%)												
1	4	(1.4)	4	(1.5)	0	(0.0)	0.008	15	(3.5)	0	(0)	< 0.001
2	103	(37)	101	(39)	2	(11)		218	(50)	3	(4.8)	
3	113	(41)	103	(40)	10	(53)		147	(34)	25	(40)	
4	15	(5.4)	14	(5.4)	1	(5.3)		22	(5.1)	4	(6.5)	
5	43	(16)	37	(14)	6	(32)		30	(6.9)	30	(48)	
Pathologic stage, *n* (%)												
T2	171	(62)	166	(64)	5	(26)	< 0.001	215	(50)	3	(4.8)	< 0.001
T3a	83	(30)	76	(29)	7	(37)		180	(41)	20	(32)	
T3b/4	24	(8.6)	17	(6.6)	7	(37)		40	(9.2)	39	(63)	
Positive surgical margins, *n* (%)	79	(28)	70	(27)	9	(47)	0.068	103	(24)	40	(48)	< 0.001
Median lymph node removed, *n* (IQR)	18	(14–24)	18	(14–24)	17	(14–22)	0.85	15	(10–20)	17	(13–24)	0.02
Median positive lymph nodes, *n* (IQR)	0	(0–0)	–		1	(1–2)	–	–		1	(1–2)	-

^†^^,‡,§,¶,††,‡‡^Corresponds to variables of nomograms listed in Table 1

### External validation of the Briganti 2019 nomogram

**Fig. 1:**
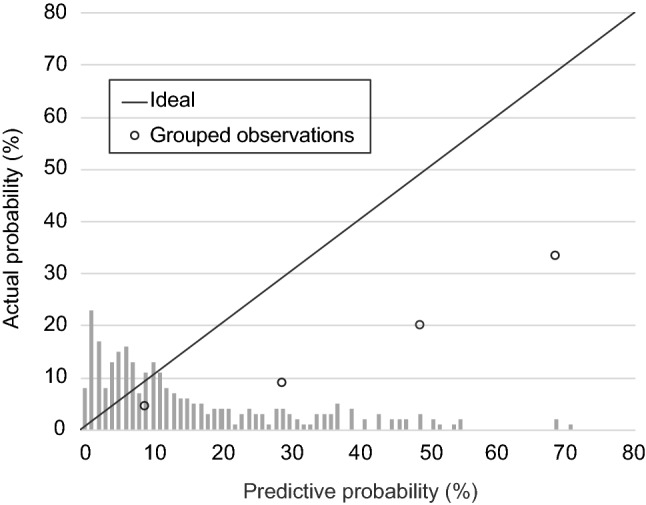
Nomogram calibration plot. The dashed line indicates the location of the ideal nomogram, in which predicted and observed probabilities are identical

As shown in Table 3, the AUC for the Briganti 2019 nomogram in our overall cohort was 0.71. On the calibration plot, the 45° line indicates perfect congruity between the predictive probability and observed value, and overestimation was noted throughout the ranges of the predicted risk of LNI (Fig. 1). Table 4 shows nomogram-derived LNI probabilities. With a cutoff set at 7%, ePLND could be omitted for 84 (30.2%) patients, and only 1 (1.2%) patient who actually had LNI would be missed. Sensitivity, specificity, and negative predictive value at a 7% cutoff were 94.7, 32.0, and 98.8%, respectively.

**Table 3:** AUCs of the nomograms in the original and external validation cohorts

Nomogram	Original cohort	Our cohort		Gandaglia et al. [21]	Diamond et al. [21]	Oderda et al. [22]
		Total	Below cT3			
Briganti 2019 [7]	0.86	0.71	0.75	0.79	0.80	0.76
Briganti 2017 [16]	0.91	0.72	0.80	0.75	N/A	0.8
Briganti 2012 [17]	0.88	0.74	0.82	0.65	0.80	0.83
MSKCC [18]	0.85	0.73	0.80	0.74	N/A	0.83
Partin 2017 [19]	0.92	–	0.73	N/A	N/A	0.79
Japan PC table [20]	0.86	–	0.79	N/A	N/A	N/A

**Table 4:** Systematic analyses of nomogram-derived cutoffs of the externally validated nomograms

Briganti 2019 [7]	Number of patients, *n* (%)												NPV
	Below the cutoff (ePLND not recommended)						Above the cutoff (ePLND recommended)						
Cutoff	Total		Without hLNI^§§^		With hLNI		Total		Without hLNI		With hLNI^¶¶^		
2	8	(2.9)	8	(3.1)	0	(0)	270	(97.1)	251	(96.9)	19	(100)	100
3	30	(10.8)	30	(11.6)	0	(0)	248	(89.2)	229	(88.4)	19	(100)	100
4	48	(17.3)	48	(18.5)	0	(0)	230	(82.7)	211	(81.5)	19	(100)	100
5	56	(20.1)	56	(21.6)	0	(0)	222	(79.9)	203	(78.4)	19	(100)	100
6	69	(24.8)	68	(26.3)	1	(5.3)	209	(75.2)	191	(73.7)	18	(94.7)	98.6
7	84	(30.2)	83	(32.0)	1	(5.3)	194	(69.8)	176	(68.0)	18	(94.7)	98.8
8	100	(36.0)	98	(37.8)	2	(10.5)	178	(64.0)	161	(62.2)	17	(89.5)	98.0
9	113	(40.6)	110	(42.5)	3	(15.8)	165	(59.4)	149	(57.5)	16	(84.2)	97.3
10	120	(43.2)	117	(45.2)	3	(15.8)	158	(56.8)	142	(54.8)	16	(84.2)	97.5
15	170	(61.2)	164	(63.3)	6	(31.6)	108	(38.8)	95	(36.7)	13	(68.4)	96.5
20	195	(70.1)	187	(72.2)	8	(42.1)	83	(29.9)	72	(27.8)	11	(57.9)	95.9
25	211	(75.9)	201	(77.6)	10	(52.6)	67	(24.1)	58	(22.4)	9	(47.4)	95.3
30	226	(81.3)	213	(82.2)	13	(68.4)	52	(18.7)	46	(17.8)	6	(31.6)	94.2
40	251	(90.3)	237	(91.5)	14	(73.7)	27	(9.7)	22	(8.5)	5	(26.3)	94.4
50	266	(95.7)	250	(96.5)	16	(84.2)	12	(4.3)	9	(3.5)	3	(15.8)	94.0
60	275	(98.9)	257	(99.2)	18	(94.7)	3	(1.1)	2	(0.8)	1	(5.3)	93.5
70	275	(98.9)	257	(99.2)	18	(94.7)	3	(1.1)	2	(0.8)	1	(5.3)	93.5
80	278	(100)	259	(100)	19	(100)	0	(0)	0	(0)	0	(0)	93.2

Nomogram	Number of patients, *n* (%)												NPV
	Below the cutoff 7%						Above the cutoff 7%						
	Total		Without hLNI^§§^		With hLNI		Total		Without hLNI		With hLNI^¶¶^		
Briganti 2019 [7]	84	(30.2)	83	(32.0)	1	(5.3)	194	(69.8)	176	(68.0)	18	(94.7)	98.8
Briganti 2017 [16]	40	(14.4)	39	(15.1)	1	(5.3)	238	(85.6)	220	(84.9)	18	(94.7)	97.5
Briganti 2012 [17]	33	(11.9)	33	(12.7)	0	(0)	245	(88.1)	226	(87.3)	19	(100)	100
MSKCC [18]	34	(12.2)	34	(13.1)	0	(0)	244	(87.8)	225	(86.9)	19	(100)	100
Partin 2017 [19]	121	(58.5)	118	(60.5)	3	(25.0)	86	(41.5)	77	(39.5)	9	(75.0)	97.5
Japan PC table [20]	97	(46.9)	96	(49.2)	1	(8.3)	110	(53.1)	99	(50.8)	11	(91.7)	99.0

^§§^Percentage is indicative of specificity

^¶¶^Percentage is indicative of sensitivity

External validation of the Briganti 2019 Nomogram compared to the other currently available nomograms.

The AUCs for the other nomograms are shown in Table 3. The AUC of Briganti 2019 in our overall cohort was confirmed to be the lowest, compared with 0.72, 0.74, and 0.73 for the Briganti 2017, Briganti 2012, and MSKCC nomograms, respectively. However, the differences were not statistically significant. For nomograms that can evaluate cases below cT3, the AUC was 0.73 for Partin 2017 and 0.79 for the Japan PC table, compared with the AUC of 0.75 for the Briganti 2019 nomogram under the same condition. As shown in Table 4, when compared using a cutoff of 7% for the Briganti 2019, Briganti 2017, Briganti 2012, and MSKCC nomograms that can evaluate the cases that include cT3, the Briganti 2019 nomogram could omit the highest number of patients (30.2% vs 14.4% vs 11.9% vs 12.2%) while limiting the number of cases of LNI that could be missed (1.2% vs 2.5% vs 0% vs 0%).

## Discussion

In our external validation, the AUC of the Briganti 2019 nomogram was the lowest compared with that of the other nomograms calculated under the same conditions. There are currently three reports of external validations of the Briganti 2019 nomogram [21–23], all of which are from Europe and include a common author. Table 3 shows the AUCs of the nomograms calculated with their cohorts. Notably, Oderda et al. highlighted a similar trend in their study, where the lowest AUC was obtained from the Briganti 2019 nomogram when compared to the other validated nomograms [23]. In addition, the AUCs of all nomograms calculated in our cohort were relatively low compared to the original and the other external validation cohorts. The calibration plot of the Briganti 2019 is below the ideal line, which indicates that the predicted probability of LNI as determined by the nomogram is overestimated compared to the actual probability. Meanwhile, sensitivity and specificity were not significantly improved if the cutoff point was changed. Therefore, changing the cutoff could not compensate for the overestimation. However, for nomograms aimed at reducing unnecessary lymph node dissection while minimizing missed lymph node metastases, the Briganti 2019 nomogram performed relatively better than other nomograms capable of assessing cases of cT3 when the cutoff was set at 7%.

The differences in the patient characteristics between the original cohort, other external validation cohort, and our cohort need to be considered. In comparison with the original cohort and our cohort, our cohort had a higher PSA and smaller prostate volume, while the maximum diameter of the index lesion on MRI was similar. Also, regarding the diagnostic accuracy of MRI to determine cT3 or higher, clinical stage tended to be underestimated compared to pathological stage in all of the studies, but the degree was the smallest in our cohort. As for the pathology results, our cohort was in a higher Gleason grade group for biopsies compared to the original cohort and other external validations. Meanwhile, when comparing the biopsy results with the final results of the surgical specimen, the other reports all tended to be upgraded while our cohort was likely to downgraded, and these tendencies in diagnostic technique may have been assumed to influence the results. In fact, between our cohort and the original cohort, the percentages of patients below the cutoff set at 7% were 30.2% and 57%, respectively, which reflect a relatively higher score in our validation cohort. Meanwhile, the results above also suggested that preoperative evaluation in our cohort tended to be overestimated, which seemed to result in low AUCs of the nomograms and a gentle gradient in the calibration plot of the Briganti 2019 nomogram. It should be noted that, in terms of the detection ability of LNI, although there were differences in surgical techniques of prostatectomy between the cohorts, the median number of lymph nodes removed was comparable, suggesting that the quality of lymph node dissection in our study was well established.

When multiple parameters were used to measure AUC, the highest AUCs for biopsy pathology were obtained using the total biopsy results rather than the targeted ones. The Gleason grade of the target lesion did not necessarily reflect cancer progression. Rather, a higher Gleason grade obtained from a systematic biopsy may reflect the extension of high-grade cancer beyond the target lesion, which could be considered as a more significant risk factor of lymph node metastasis. This may explain the lower AUC of the Briganti 2019 nomogram compared to the other nomograms, and is supported by the fact that 28 (10.1%) of the 278 cases in our cohort exhibited negative systematic biopsies, none of which were positive for LNI. Although MRI-targeted biopsy is quite useful as a diagnostic tool to increase the accuracy of cancer detection, combined MRI-targeted and systematic biopsy was considered more appropriate to determine the required therapeutic strategy.

Meanwhile, a positive systematic biopsy around the target lesion, the larger diameter of the index lesion, and higher clinical stage on MRI should have similar implications in terms of the presence of extensive malignant findings, depending on the accuracy of the imaging diagnosis of the target lesion. The diameter of the index lesion and clinical stage on MRI, both used as parameters in Briganti 2019 for positive and negative LNI, were not statistically significant. This could be due to the fact that systematic biopsies may reflect malignancies further from the target lesion, whereas our study measured the maximum diameter of the index lesion only at coronal sections, which may not accurately reflect the total tumor volume (the Briganti 2019 nomogram did not specify how the measurement was performed in the original cohort). In addition, the rate of positive LNI in cases in which pT3b was diagnosed in surgical specimens was 29.2% (7/24 cases), which was significantly higher than the 9.8% (25/254 cases) of cases with pT3a or lower. There was only one case in which the imaging and pathological diagnoses of seminal vesicle invasion were in agreement. Notably, the modified AUC of the Briganti 2019 nomogram, calculated by replacing clinical stage with pathological stage in our cohort, was increased to 0.76. An accurate assessment of the clinical stage on MRI may be key to predicting LNI.

Our study only included cases diagnosed by MRI-targeted biopsy. Cases in which the target lesion could not be identified by MRI or those in which the cancer could not be identified from the target lesion were excluded. Future studies including such cases are required.

Other parameters also remain to be studied in the future. Sato et al. reported that prostate cancer located in the anterior are less aggressive than those located in the posterior [24]. A study at our institution also suggested that cases with negative digital rectal examinations (DRE), i.e., ventral lesions, were relatively slow to progress compared to cases with positive DRE, i.e., dorsal lesions (Yoshitomi 2020, unpublished data), and further investigation regarding the relationship between the location of the tumor and its aggressiveness would be required.

Limitations of our study should be noted. First, this was a single-institute study with relatively small sample size and number of events, limiting its generalizability. Gandaglia et al. highlighted the need to generalize their nomogram to other races, stating that their cohort including mainly Caucasian males was one of the limitations of their study [7]. The majority of our cohort was Japanese, which may similarly be considered as a limitation. However, since this is the first study to include an Asian cohort when compared to all of the external validations to date, it helps to address the limitation of the original study. Also, we did not evaluate the quality of the surgical technique and the outcomes of the disease, which was not the main purpose of this study; we only evaluated the number of lymph node removed. Finally, the retrospective nature of our study is another limitation.

In conclusion, we externally validated the 2019 Briganti nomogram for the selection of patients with high-risk prostate cancer in a different cohort. Further research is warranted to identify more accurate decision-making tools, aimed at reducing unnecessary invasion while accurately identifying relevant patients and minimizing the number of LNI missed.

## Abbreviations

AUC: Area under curve
csPC: Clinically significant prostate cancer
DRE: Digital rectal examination
EAU: European Association of Urology
ePLND: Extended pelvic lymph node dissection
hLNI: Histologically confirmed lymph node invasion
IQR: Interquartile range
LNI: Lymph node invasion
mpMRI: Multi-parametric magnetic resonance imaging
MRI: Magnetic resonance imaging
MSKCC: Memorial Sloan Kettering Cancer Center
N/A: Not applicable
NPV: Negative predictive value
PC: Prostate cancer
PLND: Pelvic lymph node dissection
PSA: Prostate specific antigen
ROC: Receiver operating characteristic
RP: Radical prostatectomy
